# Friction Stir Welding of Dissimilar Al 6061-T6 to AISI 316 Stainless Steel: Microstructure and Mechanical Properties

**DOI:** 10.3390/ma16114085

**Published:** 2023-05-30

**Authors:** Mohamed Newishy, Matias Jaskari, Antti Järvenpää, Hidetoshi Fujii, Hamed Ahmed Abdel-Aleem

**Affiliations:** 1 Welding and NDT Department, Central Metallurgical Research & Development Institute CMRDI, Cairo 11421, Egypt; hamedaa@gmail.com; 2 Future Manufacturing Technologies (FMT) Research Group, Kerttu Saalasti Institute, University of Oulu, 85500 Nivala, Finland; matias.jaskari@oulu.fi (M.J.); antti.jarvenpaa@oulu.fi (A.J.); 3 Joining and Welding Research Institute, Osaka University, 11-1 Mihogaoka, Osaka 567-0047, Ibaraki, Japan; fujii@jwri.osaka-u.ac.jp

**Keywords:** friction stir welding, AISI 316 stainless steel, AA6061-T6 aluminum alloy, mechanical properties, EBSD

## Abstract

The friction stir welding (FSW) process was recently developed to overcome the difficulty of welding non-ferrous alloys and steels. In this study, dissimilar butt joints between 6061-T6 aluminum alloy and AISI 316 stainless steel were welded by FSW using different processing parameters. The grain structure and precipitates at the different welded zones of the various joints were intensively characterized by the electron backscattering diffraction technique (EBSD). Subsequently, the FSWed joints were tensile tested to examine the mechanical strength compared with that of the base metals. The micro-indentation hardness measurements were conducted to reveal the mechanical responses of the different zones in the joint. The EBSD results of the microstructural evolution showed that a significant continuous dynamic recrystallization (CDRX) occurred in the stir zone (SZ) of the Al side, which was mainly composed of the weak metal, Al, and fragmentations of the steel. However, the steel underwent severe deformation and discontinuous dynamic recrystallization (DDRX). The FSW rotation speed increased the ultimate tensile strength (UTS) from 126 MPa at a rotation speed of 300 RPM to 162 MPa at a rotation speed of 500 RPM. The tensile failure occurred at the SZ on the Al side for all specimens. The impact of the microstructure change in the FSW zones was significantly pronounced in the micro-indentation hardness measurements. This was presumably attributed to the promotion of various strengthening mechanisms, such as grain refinement due to DRX (CDRX or DDRX), the appearance of intermetallic compounds, and strain hardening. The aluminum side underwent recrystallization as a result of the heat input in the SZ, but the stainless steel side did not experience recrystallization due to inadequate heat input, resulting in grain deformation instead.

## 1. Introduction

Friction stir welding (FSW) is a green welding technology with high quality and low cost. The FSW process uses a non-consumable rotating tool with a designed shoulder and pin inserted into a butting edge or overlapped sheets or plates to be joined and traversed along the line of the joint [1,2,3,4,5]. FSW is of interest in numerous fields of application, such as the automotive, railway, air transport, aerospace, and nuclear industries [6,7,8,9]. The welding schematic of FSW can be found everywhere [10,11,12]. Since FSW is a solid-state welding process, the plastic flow is the controlling factor in the formation and the quality of the friction-stir-welded (FSWed) joints. Furthermore, the FSW technique outperforms the traditional fusion welding processes to join dissimilar and difficult-to-weld materials, such as Al/steel [13], Al/Ti [14], Mg/Steel [15], Al/Mg [16], and Al/Cu [17] combinations. However, the industrial joining of dissimilar materials remains challenging due to the need for expensive tools and sensitive operating parameters, which could affect the joint quality.

The welding of aluminum to steel remains an enormous challenge. Due to the large differences in physical and mechanical properties between the two metals, producing a sound joint of aluminum and steel with high strength has been difficult. The solubility of iron (Fe) in the Aluminum (Al) matrix is limited, and the formation of brittle intermetallic compounds (IMCs) is a serious problem. From previous literature, and from the analysis of the binary phase diagram of Al–Fe, it has been reported that during the welding of aluminum alloy and steel, brittle IMCs of Fe_2_Al_5_, FeAl_2_, FeAl, and Fe_3_Al easily form at the weld interface. The IMCs result in the poor strength of the welded joints [18,19,20,21,22].

From the open literature, FSW of Al with CS has been reported in several publications. For instance, Abbasi et al. [23] studied the FSW of low-carbon steel sheets to aluminum Al 5186 alloy 3 mm sheets. Their study reveals iron-rich fragments in the Al/Fe friction stir nugget. These fragments originated from the Fe side and traveled to the Al side. Beygi et al. [24] investigated the effect of welding parameters on the morphology, thickness, and composition of IMCs formed during the dissimilar FSW of low-carbon steel (CS) to 1050 commercially pure Al alloy. They reported that the thickness of IMCs increases with an increase in the rotation speed.

The joint properties of dissimilar FSWed mild steel to A7075-T6 aluminum alloy were investigated by Tanaka et al. [25]. The joint strength increased with the reduction in thickness of the intermetallic compound at the weld interface in agreement with Picot and Kundu [26,27]. They also announced that the joint strength increased exponentially with a decrease in the IMC thickness.

Elrefaey et al. [28] successfully welded a lap joint of pure aluminum to CS by the FSW process. They found that a slight difference in pin depth significantly affects the performance of the lap joints. Chen et al. [29] successfully FSWed Al 6111-T4 and steel DC04 sheets with no intermetallic formation with a cycle time <1 s using a novel approach, “Abrasion circle friction spot welding”. Dehghani et al. [30] studied the FSW dissimilar joining of Al 5186 to mild steel. They found that the tunnel defect was promoted and the joints’ tensile strength sharply decreased at low welding speeds, due to the formation of the thick IMCs Al_6_Fe and Al_5_Fe_2_ in the joints. They noticed that by increasing the welding speed, the IMCs decreased and the joints exhibited higher tensile strength. Campanella et al. [31] studied the feasibility of FSW dissimilar lap joining of Al 6016 and DC05 steel to be used as car-body parts. They successfully achieved sound joints. Watanabe et al. [32] successfully welded Al 5083 to SS400 mild steel by FSW. The maximum tensile strength of the joint was about 86% of that of the Al base metal (BM). Many fragments of the steel were scattered in the aluminum alloy matrix. Chen et al. [33] successfully welded dissimilar butt joints of Al 6061 aluminum alloy and SS400 low-carbon steel by FSW. They reported that lower transverse and rotation speeds yielded a higher Charpy impact value and an acceptable quality of tensile strength. Tang et al. [34] studied the effect of preheating treatment on the temperature distribution and material flow during FSW of Al 6061 and steel E235A butt joints. They reported that preheating reduces the temperature difference between the steel and the aluminum alloy, which improves the plastic flow of the two materials. As reported by Dehghani et al. [35], a continuous layer of IMC at the aluminum/steel faying surface was found to be Al_5_Fe_2_ during FSW of Al 3003-H18 to mild steel, while Al_6_(Fe, Mn) IMC was found in the weld nugget as scattered particles. The researchers obtained defect-free butt joints. The UTS of the joint increased to ~60% of that of the Al BM.

Regarding the combination of aluminum and high-strength steel, this issue has been explored in some studies [19,36,37,38,39,40,41]. In the welding of Aluminum/high-strength steel, tool rotation speed and traverse speed have significant influence on the thickness of the IMC layer formed and hence on the joint strength. Low joint strength is attributed to the formation of thicker IMCs at low tool rotation speeds and high traverse speeds. The joint strength increased with a reduction in IMC thickness. It is reported that reducing the FSW traverse speed improves the mechanical properties. Joint strength and failure position were determined by IMC thickness and stress concentration at the welded interface. In FSW of Al/high-strength steel, the common IMCs formed at the joint interface are FeAl, FeAl_2_, FeAl_3_, Fe_2_Al_5_, and Fe_3_Al.

In the continuous development of hybrid structure components, the need for the joining of aluminum alloys to stainless steel (SS) is a great challenge. Much research has been conducted to study the possibility and feasibility of FSW as a green process to weld aluminum alloys to stainless steel [42,43,44,45,46,47,48,49,50,51]. Sound joints were obtained in different combinations of Al/SS, for example, pure Al/AISI 304 [43], Al 5083/AISI 316L [44,45,46], 5052/AISI 304 [47], Al 5050/AISI 304 [20], and (Al 1100, Al 1060, and Al 2219)/AISI 321 [49,50,51]. It has been found that the diffusion of Fe, Cr, and Ni is substantial within Al; however, the diffusion of Al within SS is limited. It was reported that the change in aluminum structure is governed by a continuous dynamic recrystallization, while the microstructure changes of the SS are governed by a discontinuous dynamic recrystallization due to the low temperatures of the process. The formation of FeAl_3_, Fe_2_Al_5_, FeAl, Fe_4_Al_13_, Fe_3_Al, and Fe_2_AlCr IMCs, as well as SS fragments, was observed at the nugget interface and at the Al SZ.

The stainless steels AISI 304 and AISI 316 are popular stainless steels used in different industrial sectors due to their good mechanical properties and corrosion resistance in addition to their lower cost. Joining AISI 304 and AISI 316 stainless steels to aluminum alloys remains a great challenge. Some previous studies concerning this combination focused on the microstructure evolution in different joining configurations. In butt joining configuration, some investigations dealt with the relation between mechanical strength and the existence of stainless steel particles in the aluminum or on the IMC growth following the main process parameters [50,51,52,53,54,55,56,57,58,59,60,61,62,63].

Jabraeili et al. [50] studied the effect of FSW parameters on the microstructure and mechanical properties of the dissimilar butt joints of Al 2024/AISI 304 SS. They found that the tool offset had a crucial effect on the microstructure and mechanical properties of the joint, since tool offset controls the HI which influences the IMCs formed. During FSW, a composite structure composed of dynamically recrystallized aluminum matrix and ultra-fine grains of SS fragments surrounded by IMCs forms in the SZ. Goel et al. [51] obtained joints with a UTS ~71% of that of the Al BM in dissimilar FSW of Al 7475-T761/AISI 304 SS. They also found that tool offset is critical to the success of joints, in agreement with Jabraeili et al. [50]. Muhamad et al. [52] investigated the effect of the addition of Al-Ni powder on the performance of Al 7075-T6/AISI 304L FSWed butt joints. They found that the Al-Ni powder addition increased the tensile strength due to the increased contact at the interface during lower tool rotational speeds. Zhang et al. [53] obtained high-quality Al 1060Al/AISI 304 SS joints by FSW with no contact between the tool and the steel plate using an ordinary H13 steel tool. A joint with a high UTS of ~95% of that of the Al BM was achieved. Ogura et al. [54] investigated the mechanical and metallurgical properties of FSWed Al 3003/AISI 304 SS dissimilar lap joining. Sound joints were obtained. Hatano et al. [55] investigated the influence of the IMC layer thickness for Al 6061/AISI 304 SS dissimilar joint strength. They found that joints without heat treatment showed interfacial fractures due to the oxide film. Heat treatments affect the IMC layer thickness and improve dissimilar joint strength. Harwani et al. [56] studied the effect of shoulder diameter on dissimilar butt joints for Al 6061/AISI 304 SS. They found that the tensile strength, percentage elongation, and joint efficiency increased marginally with the increase in the shoulder diameter. A maximum UTS ~31% of that of the Al BM was achieved. Uematsu et al. [57] investigated the fatigue crack propagation (FCP) of FSWed Al 6061-T6/AISI 304 SS joints. Compared with the Al alloy BM, the FSWed joints exhibited slower FCP rates with higher FCP resistances than the BM. When studying the same materials, Kakiuchi et al. [58] found that the FCP rates of the Al/SS FSWed joint were comparable or slightly faster than those of the Al BM, in contrast to the results of Uematsu et al. [57]. Uzun et al. [59] studied the fatigue properties of FSWed dissimilar Al 6013-T4/AISI 304 SS joints. The fatigue properties of the FSWed joints were found to be approximately 30% lower than those of the Al BM, in agreement with Uematsu et al. [57]. Bang et al. [60] evaluated the potential of using the gas tungsten arc welding (GTAW) assisted hybrid friction stir welding (HFSW) process to join Al 6061 Al/AISI 304 SS, in order to improve the weld strength. A UTS ~93% of that of the Al BM was obtained.

The improved UTS may be due to the enhanced material plastic flow and partial annealing effect in dissimilar materials due to the preheating of the SS surface by GTAW, resulting in the significantly increased elongation of welds. On the other hand, with the softening of the SZ, the hardness decreased about 50% in regard to the aluminum alloy BM. Paventhan et al. [61] welded AA6082/AISI 304 SS dissimilar joints by FSW. They reported an empirical relationship to predict the tensile strength of friction-welded joints; when incorporating the process parameters effectively, their method was used to predict the tensile strength of friction-welded joints at a confidence level of 95%. Li et al. [62] studied the effect of a high rotational speed (HRS) of 10,000 RPM and an ultra-high rotational speed (ultra-HRS) of 18,000 RPM on the FSW of 1.0 mm thickness Al 6061-T6 to AISI 316L SS with 0.8 mm thickness. It was observed that the formation of intermetallic compounds is solely related to the rotational speed. The maximum tensile strength was found to be about 75.9% of that of the Al BM. Zandsalimi et al. [63] investigated the effect of FSW parameters on the microstructure and the mechanical properties of the Al 6061/AISI 430 SS dissimilar joints. They found that the best appearance quality was achieved at a higher rotational speed and zero tool offset, while the negative and positive values of the offsets led to the formation of voids and microcracks which reduced the tensile properties of the joints. The resulting stir zone of the joints had a composite structure in which the dispatched stainless steel particles were distributed in the aluminum SZ.

Due to a lack of literature studying the FSW of AA6061 and AISI 316 SS, and since both materials are crucial combinations in many industries such as the automotive industry, the main objective of this work was to investigate the FSW dissimilar butt joining of 3 mm thickness Al 6061-T6/AISI 316 SS. In addition, extensive microstructure investigations of the SZ and the nugget interface were performed with EBSD to reveal the recrystallization and deformation in both welded materials. Mechanical properties were investigated through uniaxial tensile tests and indentation hardness tests.

## 2. Experimental Procedures

**Figure 1:**
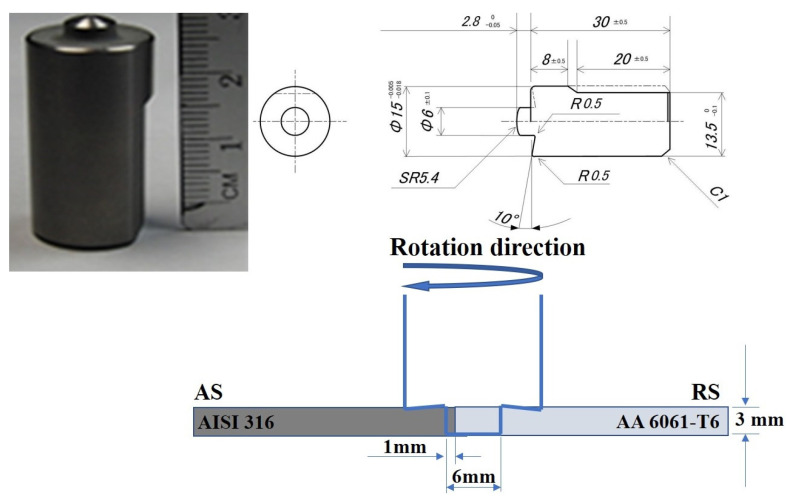
WC-Co tool general view, dimensions, and experiment schematic view.

The AISI 316SS and 6061-T6 aluminum alloy sheets with dimensions of 120 mm × 50 mm × 3 mm were selected as base metals (BMs). The chemical composition and mechanical properties of the BMs are summarized in Table 1 and Table 2, respectively. The dissimilar BMs were friction-stir-welded according to the friction stir parameters summarized in Table 3. Rotational speed, traverse speed, and tool offset were the process parameters. A tool was made of tungsten carbide–cobalt (WC-Co) consisting of a 15 mm shoulder with a 6 mm cylindrical-shaped pin. Aluminum and stainless steel sheets were placed on the retreating side (RS) and the advancing side (AS), respectively. The pin of the tool was shifted 2 mm toward the Al side. Figure 1 shows a photo of the tool used and a schematic drawing for tool dimensions and experiment setup. The tilt angle of the tool was fixed at 3° from the normal axis to the sheet’s surfaces. A water-cooled tool holder was used for the experiments. Argon shielding was employed around the tool at a flow rate of 5 × 10^5^ mm^3^ s^−1^ to avoid the surface oxidation of the friction-stir-welded zone. Load control mode was employed in the current experiments to keep the forging load of the tool constant for every experiment at 15 kN. The rotational speeds were within the range of 300 to 600 RPM and the travel speeds were 20 and 30 cm/min^−1^.

**Figure 2:**
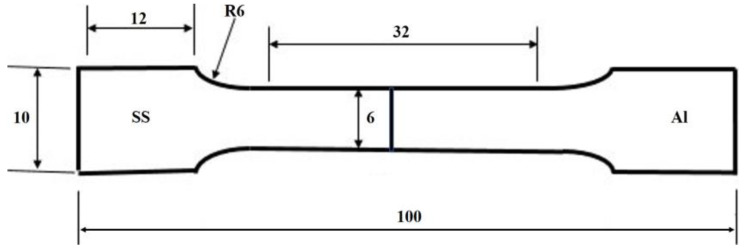
Schematic drawing of the ASTM E8/8M tensile specimen subsize.

For the metallographic examination using EBSD, the dissimilar SS/Al butt joints welded by FSW were sectioned perpendicular to the welding direction, and the transverse cross-section was metallographically prepared according to the standard preparation technique, i.e., mechanically ground using SiC papers, then ground down to 1 µm by using a diamond suspension, and finally chemically polished using a 0.05 µm colloidal suspension of silica for about 10 min. The microstructural characteristics of the fusion zone (FZ) and thermomechanical affected zone (TMAZ) were studied using a field-emission gun scanning electron microscope (FEG-SEM) (model: Carl Zeiss Ultra plus: Oberkochen, Germany) that was equipped with electron backscatter diffraction (EBSD) capability with step sizes 0.4, 0.2, and 0.1 μm for 250X, 500X, and 1000X, respectively. The weld morphologies at different FSW rotation speeds, such as mixed zone size and geometry, were investigated using a laser scanning confocal microscope (model: KEYENCE/VKX200: Osaka, Japan). In accordance with the ASTM E8/8M standard, subsize tensile specimens with a gauge length of 32 mm and width of 6 mm were then machined perpendicular to the welding direction as shown in Figure 2. Two samples of each butt joint were tensile tested for each RPM. The tensile properties of the dissimilar joints were evaluated using a Zwick Z 100 tensile machine (Zwick Roell, Ulm, Germany) at a quasi-static strain rate of 10^−3^ s^−1^. To achieve accurate measuring of the tensile properties, an external extensometer was used. Micro-indentation experiments were carried out using a calibrated micro-indenter CSM equipped with a standard Berkovich indenter. The micro-indentation hardness (H_IT_) tests were conducted by increasing the load up to a maximum force of 2 N at loading and unloading rates of 66.66 mN/s with a holding time of 15 s. The average of 5 H_IT_ measurements for each zone was used. Based on the loading–unloading cycles, the force–penetration depth (F-D) curves were obtained up to the maximum indentation force of 2 N.

## 3. Results and Discussion

### 3.1. Characteristics of the Base Metals

**Figure 3:**
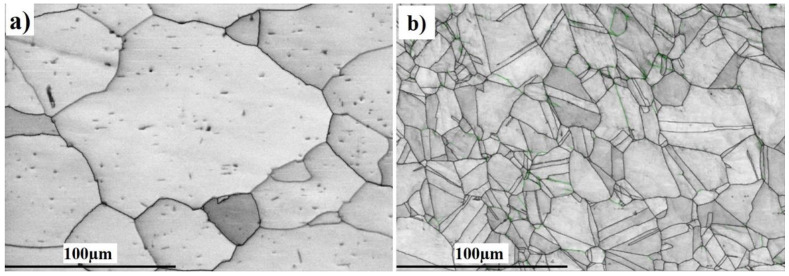
Microstructures of the base metals used: (**a**) 6060-T6 aluminum alloy, and (**b**) AISI 316 SS.

Typical microstructures of the base metals (BMs) used in processing the dissimilar friction-stir-welded butt joints are shown in Figure 3. The initial microstructures of the experimental BMs were significantly different in terms of grain morphology and grain size. It can be seen from Figure 3a that the structure of the Al BM is a typical rolled structure with an average grain size of ~78 µm. The microstructure of AISI 316 SS in Figure 3b shows a fully austenitic equiaxed grain structure including twins, sub-grains, and slip bands. Austenite grains are elongated in the direction of forming with an average grain size of ~14 µm. The engineering flow curves of the BMs, i.e., AISI 316 SS and 6061-T6 aluminum alloy, as measured using uniaxial tensile testing, are shown in Section 3.4. The ultimate tensile strength of AISI 316 SS is 650 MPa, which is twice that of the 6061-T6 aluminum alloy (290 MPa), while the yield strength of both metals is 225 MPa for aluminum and 250 MPa for stainless steel. Both metals exhibit a continuous transition from elastic to plastic behavior.

### 3.2. Surface Appearance and Macrostructure

**Figure 4:**
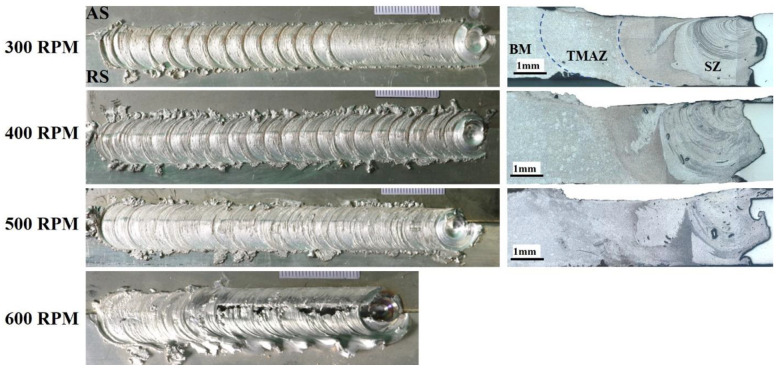
Surface appearance and corresponding cross-section of FSWed AISI 316 SS to 6061 Al alloy at different RPMs.

The appearances and a corresponding cross-sectional view of the friction-stir-welded (FSWed) 6061-T6/AISI 316 SS butt joints at 300–600 RPM are provided in Figure 4. The surface appearance of the welded joints became smoother as the rotational speed of the tool was raised from 300 to 500 RPM, and then the weld failed at a rotation speed of 600 RPM. It is well documented that rotational speed and traverse speed have a direct effect on the thermal history of the FSWed joints [52]. This is attributed to an increase in heat input due to the tool rotation speed increase. By increasing the RPM or decreasing the traverse speed, the peak temperatures during FSW increase. It is well known in the literature that a rise in the tool RPM will raise the plastic flow, and will increase the viscoplastic heat generation and the total heat input during the FSW. However, the effect of rotational speed is greater than that of the traverse speed [64,65,66].

Optical micrographs of the cross-sections that are perpendicular to the welding path of the joints at different tool RPMs are shown in Figure 4. The joint interface consists of mainly three distinct zones: namely, the stir zone (SZ), the thermomechanical affected zone (TMAZ), and the unaffected base metal (BM). No sign of a heat-affected zone (HAZ) was observed in the joint, probably due to optimal heat generation during the FSW. The volume of the SZ was nearly equal to the diameter of the shoulder near the plate surface and the diameter of the pin near the bottom side. It was also interesting that the thickness of the stirred Al material decreased in the area under the tool shoulder. It was obviously apparent that all joints were flawless and tunnel-free. This indicated that sufficient heat input was introduced during dissimilar joining, which resulted in adequate material flow and the admixture of both AA6061/AISI 316 SS alloys. It was also seen that the amount and distribution of stainless steel fragments increased and became more homogeneous by increasing the tool RPM, in agreement with the work of Ebrahimzadeh et al. [67]. The weld zones of the FSWed AA6061/AISI 316 SS joints at different rotational speeds appeared similar in terms of visual assessment.

### 3.3. X-ray Diffraction Analysis

**Figure 5:**
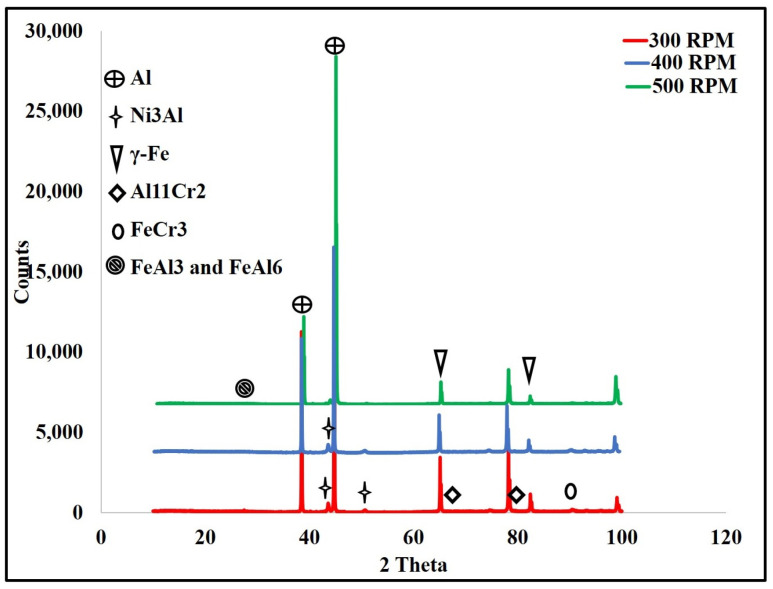
X-ray diffractometer (XRD) patterns for possible Al compounds for the specimen with tool rotation speed of 300–500 RPM.

An X-ray diffraction analysis was used to determine the aluminum/steel IMCs generated by the FSW process. Aluminum, aluminum nickel, iron, aluminum chrome, and aluminum iron intermetallic peaks were detected as shown in Figure 5. The presence of aluminum compound peaks in the X-ray pattern confirmed the occurrence of the in situ reaction between the alloying elements and the aluminum matrix forming intermetallic compounds in the particulate or fragmental conditions. The intensity of aluminum iron was considered to be at a high percentage, from 25% at 300 RPM up to 29% at 500 RPM for FeAl_3_, in addition to 14% for FeAl_6_ at 500 RPM. This result was in agreement with Li et al. [61], who obtained Fe_3_Al and FeAl_3_ IMCs along the weld interface.

### 3.4. Microstructural Characteristics of the Welded Dissimilar Joints

**Figure 6:**
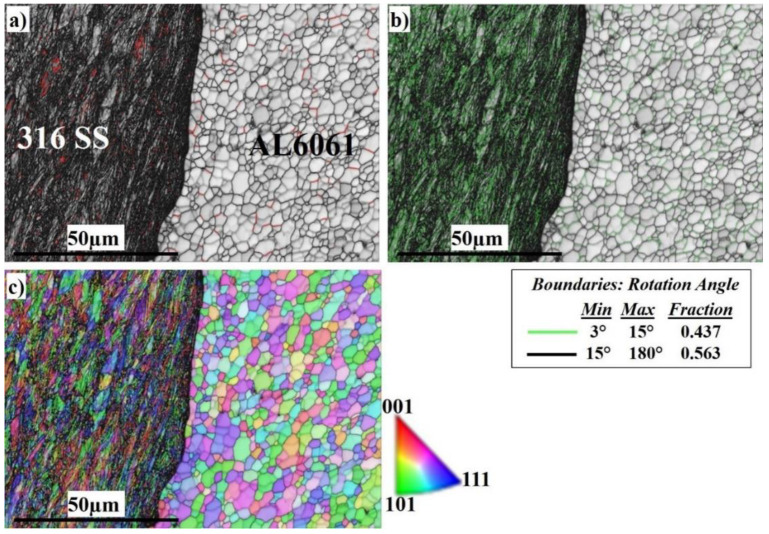
EBSD maps of the nugget interface at 300 RPM: (**a**) IQ map, (**b**) misorientation map, and (**c**) IPF color map.

**Figure 7:**
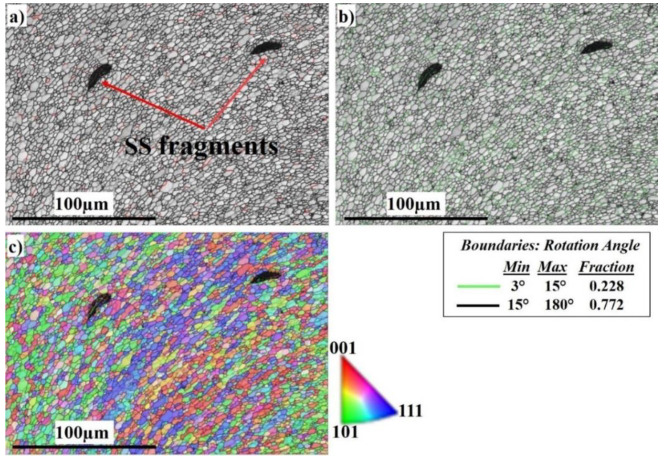
EBSD maps of the nugget in the Al 6061 side at 300 RPM: (**a**) IQ map, (**b**) misorientation map, and (**c**) IPF color map.

To analyze the effect of welding rotation speed on the microstructural features, and to disclose more details of the microstructure formation mechanism of the SZ, electron backscatter diffraction (EBSD) was employed at low and high magnifications. Figure 6 displays a general view of the microstructure of the SZ interface at 300 RPM. From the image quality (IQ) map in Figure 6a, it can be observed that the weld structure is illustrated without any major defects. The interface between Al and stainless steel is clearly sharp. The light-colored grains represent the grains of the Al 6061 on the right side and the dark-colored grains represent the grains of the AISI 316 SS on the left side. Figure 6b shows the corresponding misorientation map of all boundaries in Figure 6a. According to that map, low-angle grain boundaries (LAGBs) with a misorientation angle <15° indexed in green with a 0.437 fraction have appeared intensively on the SS side. The green boundaries are mainly the boundaries of grains and sub-grains of the austenitic matrix due to the high density of dislocations and low stacking-fault energy (SFE) [68,69]. The high-angle grain boundaries (HAGBs) with a 0.563 fraction are present mainly on the Al side with a high SFE. According to the inverse pole figure (IPF) map in Figure 6c, the DRX of the aluminum and stainless steel is obvious. The SS side shows discontinuous dynamic recrystallization (DDRX) which results in LAGBs, while the Al side shows continuous dynamic recrystallization (CDRX) which results in HAGBs, as shown in Figure 6b [70]. Figure 7 shows the EBSD imaging maps of the SZ and TMAZ of the Al side at 300 RPM. The IQ map, Figure 7a, shows the fine equiaxed recrystallized grain structures in the SZ; the SZ contains little stainless steel fragments of different sizes, which transferred from the stainless steel AS to the aluminum RS, to mix with the aluminum matrix. The SS fragments are completely coherent with the matrix with no defects. The corresponding misorientation map, Figure 7b, shows a high density of HAGBs with a misorientation angle >15° (0.772 fraction). The misorientation change across the SZ on the Al side has a typical low-energy dislocation structure [44]. Dislocation annihilation took place due to the lower viscoplasticity of aluminum compared to steel [68]. Figure 7c, the IPF color map, illustrates the different orientations of the fine recrystallized Al grains.

**Figure 8:**
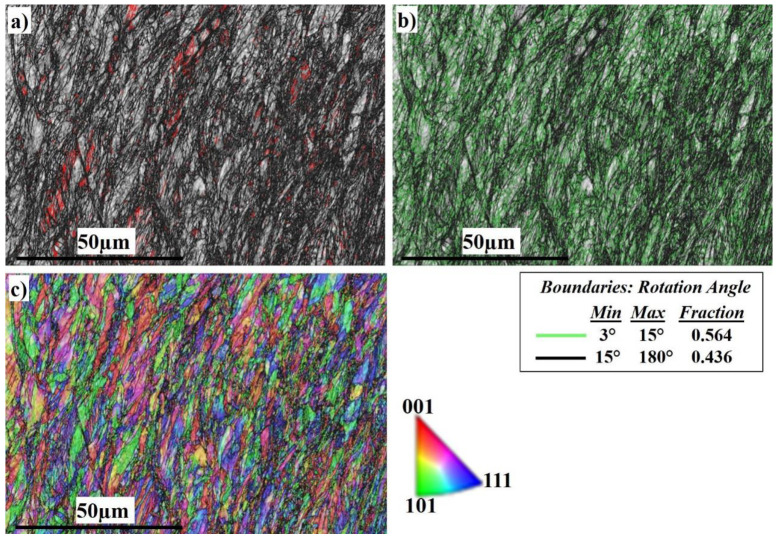
EBSD maps of the SZ of the SS side at 300 RPM: (**a**) IQ map, (**b**) misorientation map, and (**c**) IPF color map.

Figure 8 shows the EBSD imaging maps of the SZ and TMAZ of the SS side at 300 RPM. The IQ map in Figure 8a shows a very fine deformed grain structure in the SZ, while the TMAZ grain size becomes coarser. Darker regions appear on the right side which directly indicates severe plastic deformation of the highly viscoplastic stainless steel. Dark regions are regions with reduced IQ values and this indicates that a collapse of the EBSD pattern quality occurred at these regions due to higher dislocation density. A linear correlation between the level of dark regions/lines in the IQ images and the dislocation density was reported by Paidar et al. [69]. The corresponding misorientation map, Figure 8b, shows the high density of LAGB with a misorientation angle <15° (0.564 fraction) compared to (0.436 fraction) HAGBs. The misorientation change across the SZ in the stainless steel side has a typical high-energy dislocation structure. Dislocation entanglement took place due to the higher viscoplasticity of stainless steel compared to aluminum. Figure 8c, the IPF color map, illustrates the different orientations of the fine recrystallized grains.

**Figure 9:**
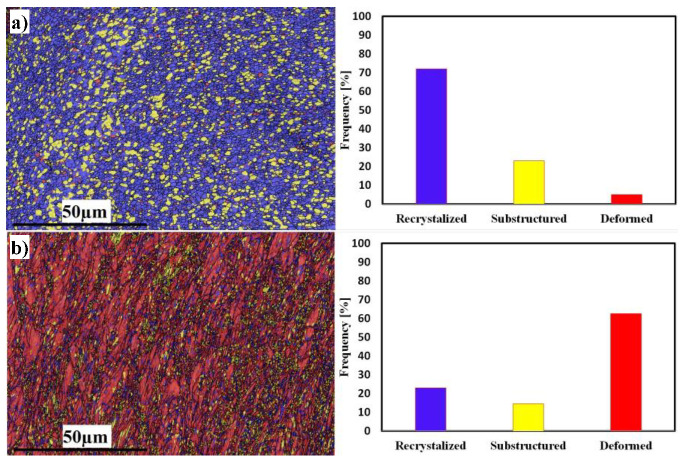
EBSD maps of recrystallized, substructure, and deformed grains; misorientation angle distributions of Al mixed zone (**a**) and SS deformed zone (**b**), at 300 RPM.

The recrystallization and deformation analyses of both sides at 300 RPM are shown in Figure 9. The Al side shows 72% grain recrystallization, 23% substructure, and 5% deformed grains (Figure 9a), while the SS side shows the opposite behavior, with 23% grain recrystallization, 15% substructure, and 62% deformed grains (Figure 9b). The behavior of SS exhibits deformed grains rather than recrystallized ones; this is due to the insufficient heat generated during FSW. The average grain sizes of the SZ in Al and SS were 4 and 1 µm, respectively.

**Figure 10:**
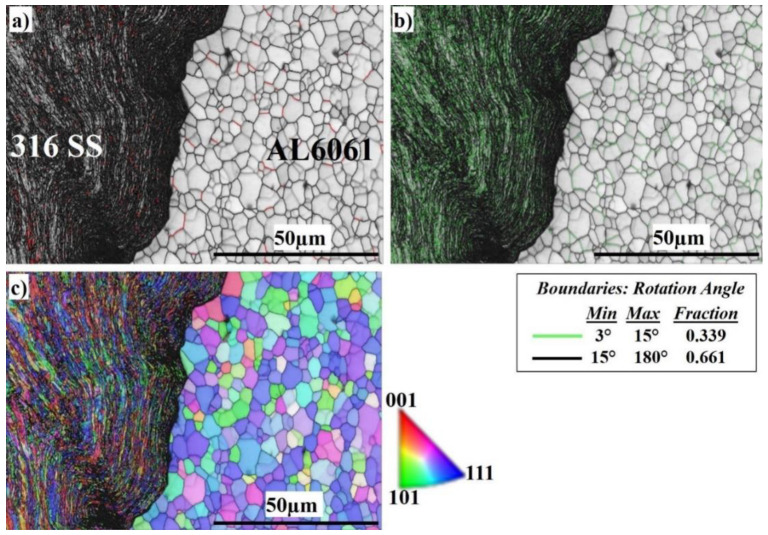
EBSD maps of the nugget interface at 400 RPM: (**a**) IQ map, (**b**) misorientation map, and (**c**) IPF color map.

A general view of the microstructure of the SZ interface at 400 RPM is shown in Figure 10. From the image quality (IQ) map in Figure 10a, it can be observed that the weld structure is illustrated without any major defects. The interface between aluminum and stainless steel is clearly sharp. The light-colored grains represent the grains of the Al 6061 on the right side and the dark-colored grains represent the grains of the AISI 316 SS BM on the left side. Figure 10b shows the corresponding misorientation map of all boundaries in Figure 9a. According to that map, LAGBs with (0.339 fraction) have appeared intensively in the austenite phase side, and are also present on the Al side. HAGBs with (0.661 fraction) are also present mainly on the aluminum side. According to the inverse pole figure (IPF) map in Figure 10c, the DRX of the Al is obvious. The SS side shows the deformed structure, which results in LAGBs, while the Al side has developed CDRX which results in HAGBs, as shown in Figure 10b.

**Figure 11:**
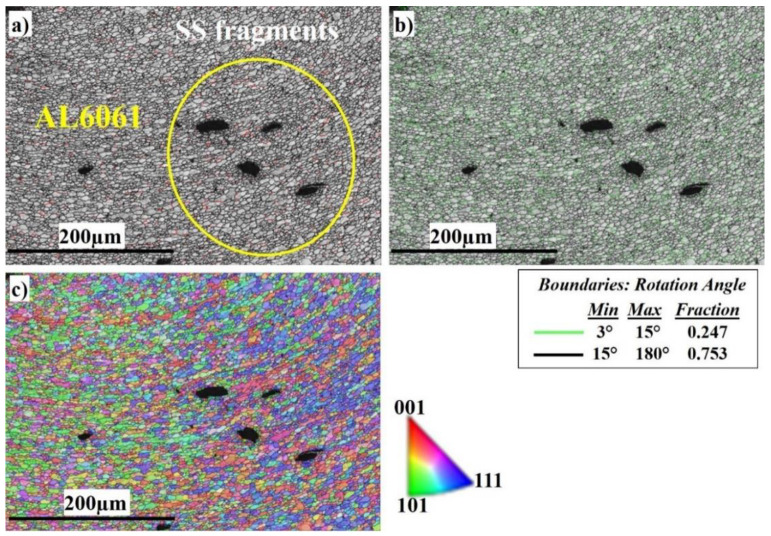
EBSD maps of the nugget center at 400 RPM: (**a**) IQ map, (**b**) misorientation map, and (**c**) IPF color map.

Figure 11 shows the EBSD imaging maps of the SZ center of the Al side at 400 RPM .

Figure 11a shows the fine equiaxed recrystallized grain structure in the SZ; the SZ contains SS fragments of different sizes (shown in the yelleow circle), which are transferred from the AS to the RS, and to the Al to mix with the Al matrix. The SS fragments are completely coherent with the matrix with no defects. The corresponding misorientation map, Figure 11b, shows a high density of HAGBs with a misorientation angle >15° (0.753 fraction). The misorientation change across the SZ on the Al side has a typical low-energy dislocation structure. The fraction of HAGBs increases compared with Figure 11b because the indexing is for Al grains only. Dislocation annihilation took place due to the higher viscoplasticity of aluminum compared to steel. Figure 11c, the IPF color map, illustrates the different orientations of the refined Al grains.

**Figure 12:**
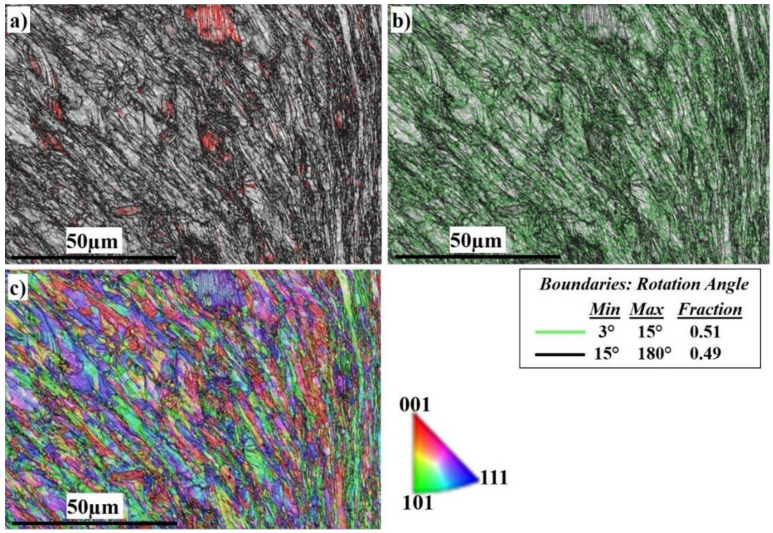
EBSD maps of the AISI316 nugget side at 400 RPM: (**a**) IQ map, (**b**) misorientation map, and (**c**) IPF color map.

Figure 12 shows the EBSD imaging maps of the SZ of the SS side at 400 RPM. The IQ map in Figure 12a shows a very fine equiaxed recrystallized grain structure in the SZ, while the TMAZ grain size becomes coarser. Darker regions appear on the right side which directly indicates severe plastic deformation of the highly viscoplastic SS. The corresponding misorientation map, Figure 12b, shows a high density of LAGB with (0.51 fraction) compared to (0. 49 fraction) HAGBs. The misorientation change across the SZ on the SS side has a typical high-energy dislocation structure. The HAGBs increase compared with those at 300 RPM; this is due to the increase in heat input which allows the structure to relieve stresses and the dislocation density to decrease. Figure 12c, the IPF color map, illustrates the different orientations of the deformed SS grains.

**Figure 13:**
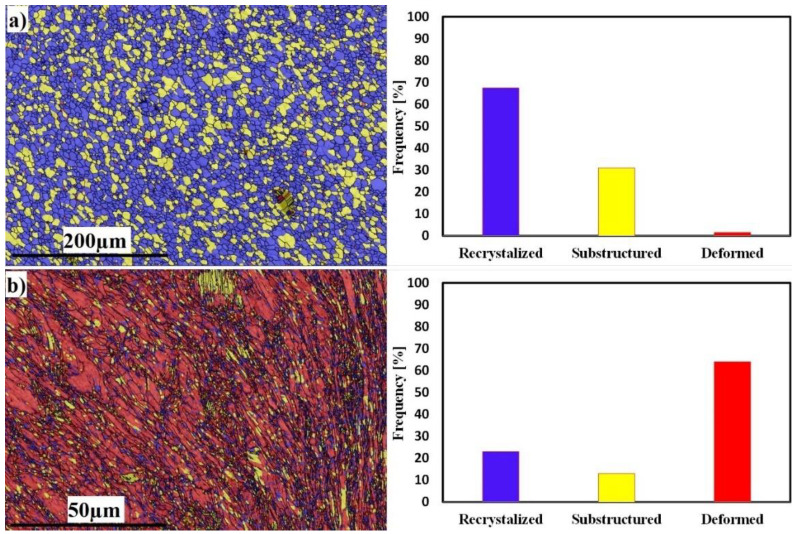
EBSD maps of recrystallized, substructure, and deformed grains; misorientation angle distributions of Al mixed zone (**a**) and SS deformed zone (**b**), at 400 RPM.

The recrystallization and deformation analyses of both sides at 400 RPM are shown in Figure 13. The Al side shows 67% grain recrystallization, 31% substructure, and 2% deformed grains (Figure 13a), while the SS side shows the opposite behavior, with 23% grain recrystallization, 13% substructure, and 64% deformed grains (Figure 13b). The behavior of SS exhibits deformed grains rather than recrystallized ones; this is due to the insufficient heat generated during FSW. The average grain sizes of the SZ in Al and SS were 6 and 1 µm, respectively.

**Figure 14:**
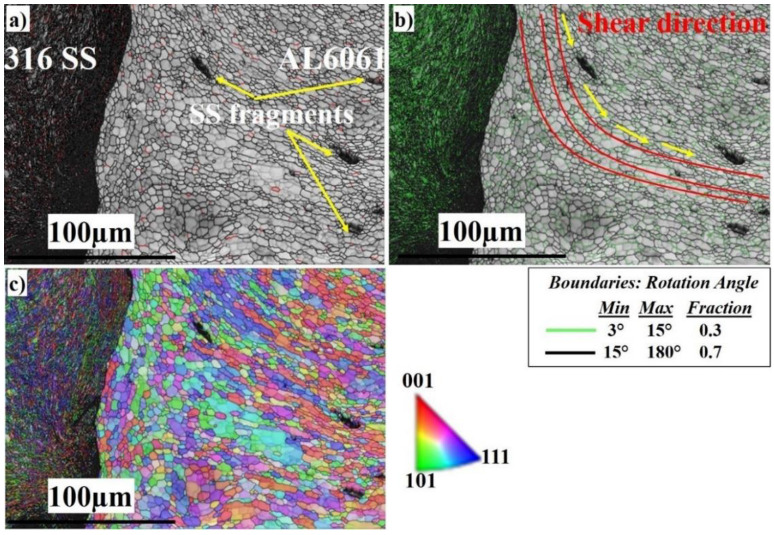
EBSD maps of the nugget interface at 500 RPM: (**a**) IQ map, (**b**) misorientation map, and (**c**) IPF color map.

A general view of the microstructure of the SZ interface at 500 RPM is shown in Figure 14. From the image quality (IQ) map in Figure 14a, it can be observed that the weld structure is illustrated without any major defects. The interface between Al and SS is clearly observed. The light-colored grains represent the grains of the Al 6061 on the right side and the darkcolored grains represent the grains of the AISI 316 SS BM on the left side. Figure 14b shows the corresponding misorientation map of all boundaries in Figure 13a. According to that map, LAGBs with (0.3 fraction) have appeared intensively in the austenite phase side and are also present on the Al side. HAGBs with (0.7 fraction) are also present mainly on the aluminum side. It appears that the shear direction is wider at 500 RPM; grain growth takes place as the heat input increases. This allows the microstructure to relieve stress and, accordingly, dislocation annihilation occurs which in turn increases the HAGBs [36,41,46,60]. According to the inverse pole figure (IPF) map in Figure 14c, the DRX of the Al is obvious. The SS side shows DDRX which results in LAGBs, while the Al side has developed continuous CDRX which results in HAGBs, as shown in Figure 14b.

**Figure 15:**
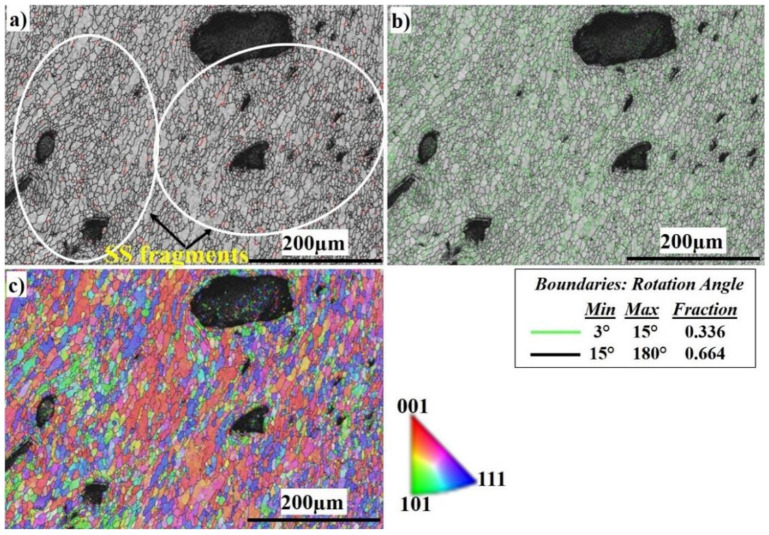
EBSD maps of the SZ center at 500 RPM: (**a**) IQ map, (**b**) misorientation map, and (**c**) IPF color map.

**Figure 16:**
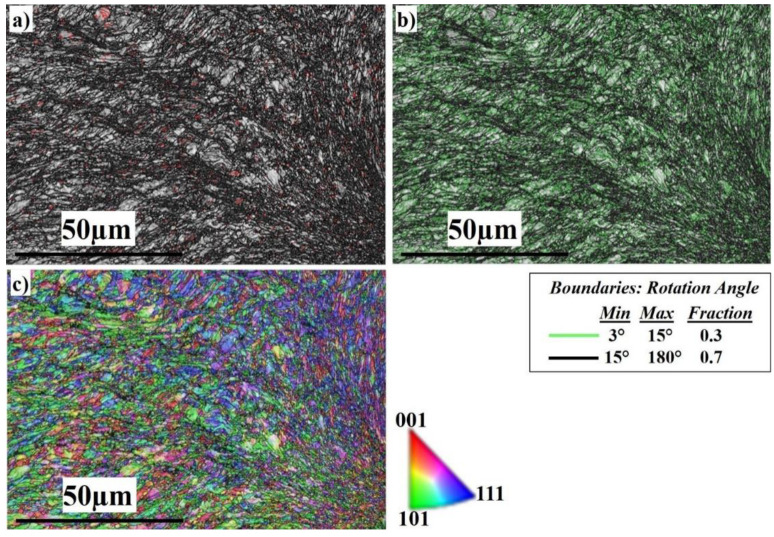
EBSD maps of the AISI316 nugget side at 500 RPM: (**a**) IQ map, (**b**) misorientation map, and (**c**) IPF color map.

Figure 15 shows the EBSD imaging maps of the SZ center of the Al side at 500 RPM. The IQ map, Figure 15a, shows the fine equiaxed recrystallized grain structure in the SZ; the SZ contains SS fragments of different sizes (shown in the white circle), which are transferred from the AS to the RS, to mix with the Al matrix. The SS fragments are completely coherent with the matrix with no defects. The SS fragments increase in amount and size at 500 RPM rotation speed. The corresponding misorientation map, Figure 15b, shows a high density of HAGBs with (0.664 fraction). The misorientation change across the SZ on the Al side has a typical low-energy dislocation structure. Figure 15c, the IPF color map, illustrates the formation of recrystallized fine grains, which are larger in size compared to those of the 300 and 400 RPM rotation speeds. Figure 16 shows the EBSD imaging maps of the SZ of the SS side at 500 RPM. The IQ map, Figure 16a, shows the fine equiaxed recrystallized grain structure in the SZ, while the TMAZ grain size becomes coarser. Darker regions appear on the right side which directly indicates severe plastic deformation of the highly viscoplastic SS. The corresponding misorientation map, Figure 16b, shows a high density of HAGB with (0.7 fraction) compared to (0.3 fraction) LAGBs. The misorientation changes across the SZ on the SS side are due to the increase in heat input compared with that at 300 and 400 RPM, which allows the structure to relieve stress and the dislocation density to decrease. Figure 16c, the IPF color map, illustrates the different orientations of the FSed SS grains.

**Figure 17:**
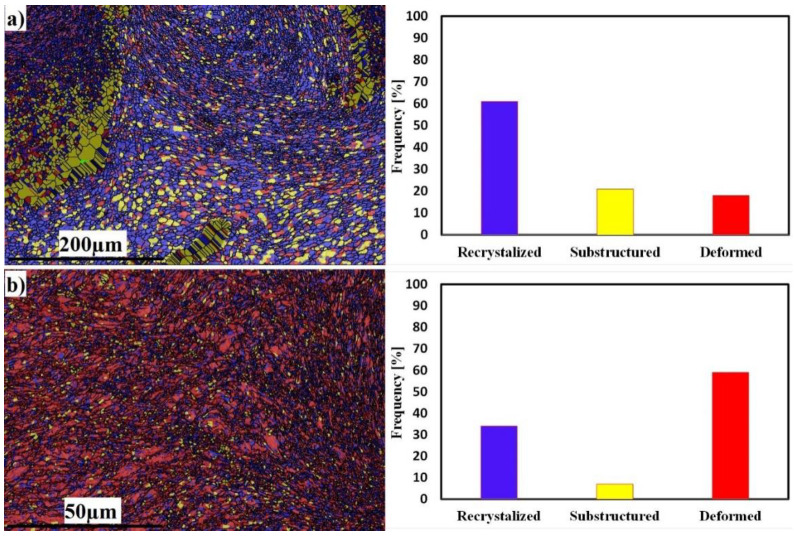
EBSD maps of recrystallized, substructure, and deformed grains; misorientation angle distributions of Al mixed zone (**a**) and SS deformed zone (**b**), at 500 RPM.

The recrystallization and deformation analyses of both sides at 500 RPM are shown in Figure 17. The Al side shows 61% grain recrystallization, 21% substructure, and 18% deformed grains (Figure 16a), while the SS side shows 34% grain recrystallization, 7% substructure, and 59% deformed grains (Figure 17b). The behavior of SS exhibits deformed grains rather than recrystallized ones, although the recrystallized grains fraction increased due to the increase in heat input. The average grain sizes of the SZ in Al and SS were 6 and 1 µm, respectively.

**Figure 18:**
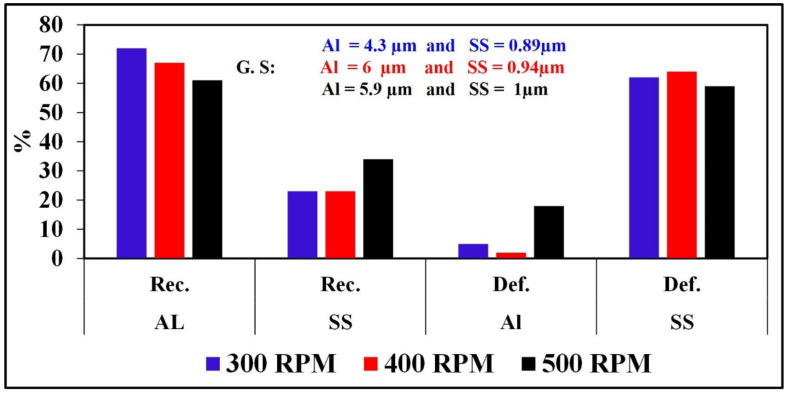
Comparison chart for % recrystallization, % deformation, and grain sizes of Al and SS at all studied FSW parameters.

Figure 18 shows a comparison chart for % recrystallization, % deformation, and grain size for Al and SS at all studied FSW parameters.

### 3.5. Tensile Properties and Micro-Indentation Hardness of Dissimilar Metal Joints

**Figure 19:**
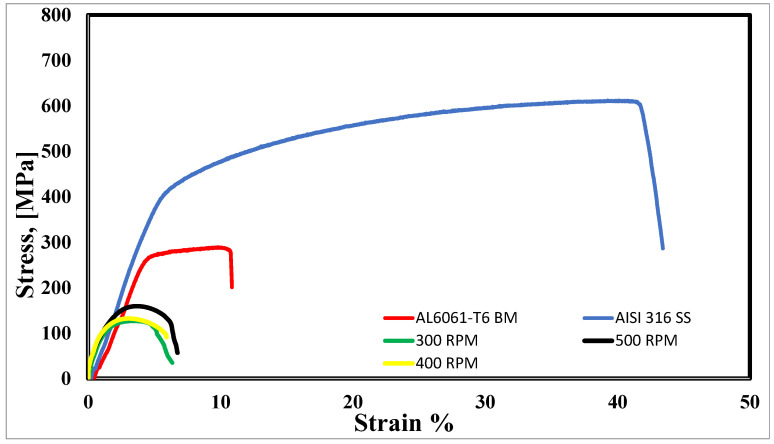
Tensile flow curves of as-received BMs and the dissimilar FSWed butt joints with different RPMs.

After cutting two tensile samples from each FSWed sheet, tensile tests were performed and compared for their UTS values. The as-received 6061-T6 plate showed a UTS of 290 MPa. The highest UTS in the FSWed samples had a UTS equal to 56% of that of the as-received Al sheet at 500 RPM and 20 cm/min traverse speed. The lower UTS of the welded samples compared to the parent metal is 44% at 300 RPM and 30 cm/min traverse speed. The low UTS for welded specimens could be attributed to the formation of intermetallic compounds in the weld interface. Figure 19 represents the engineering tensile flow stress curves of the studied dissimilar butt joints compared to the BMs at room temperature.

**Figure 20:**
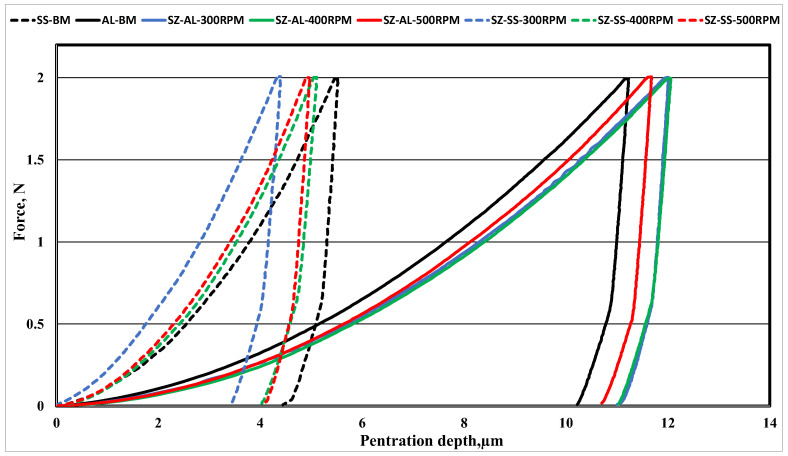
Indentation load–penetration depth curves, loading–unloading curves of BMs, and different zones of FSWed dissimilar joints at different RPMs.

The typical micro-indentation load–penetration depth (P–h) curves of the BMs and the different zones of the FSWed dissimilar joints at different RPMs are summarized in Figure 20. For instance, the average H_IT_ of the Al 6061-T6 and AISI316 SS BMs are 0.65 and 2.98 GPa, respectively. The average H_IT_ of the welded joint AISI316 SS SZ and the Al SZ at 300 RPM were 0.578 and 5.038 GPa, respectively. The average H_IT_ of the welded joint AISI316 SS SZ and the Al SZ at 400 RPM were 0.586 and 3.385 GPa, respectively. The average H_IT_ of the welded joint AISI316 SS SZ and the Al SZ at 500 RPM were 0.63 and 3.6 GPa, respectively. It is obvious that the hardness of the SZ of the Al side increases with an increase in the RPM; this is due to the grain refinement of the stirred zone in addition to the increase in the amount of SS fragments in the Al nugget which reinforces the matrix. Normally, the hardness of the Al nugget decreases compared with that of the Al BM; this is of course due to the loss of strengthening heat treatment applied to the BM before FSW, despite the effect of the grain refinement after FSW [71]. On the other hand, the SZ hardness of the SS side decreases with an increase in the rotation speed, which could be due to the decrease in dislocation densities as the heat input increases, allowing less viscoplasticity of the SS structure [72,73]. The measured H_IT_ for the TMAZ of the Al side was nearly equal to the H_IT_ of the Al BM, while the H_IT_ of the TMAZ of the SS side was nearly equal to the SS SZ.

## 4. Conclusions

In this study, the effect of FSW rotation speed on the microstructure and the mechanical properties of the dissimilar stainless steel AISI316 and aluminum alloy AA6061-T6 butt joints were investigated. The main results of this research can be summarized as follows:

1. Successful butt joints were obtained between stainless steel AISI316 and the aluminum alloy AA6061-T6.
2. At the interface of the Al and SS, many kinds of IMCs were found, including FeAl_6_, FeAl_3_, Ni_3_Al, Al_11_Cr_2,_ and FeCr.
3. Dissimilar welded butt joints show very fine recrystallized grains for Al 606 and very fine deformed grains of the AISI 316 SZ with HAGBs and LAGBs, respectively.
4. The highest obtained UTS of 160 MPa was found for the joint at 500 RPM and 20 cm/min traverse speed, which is 56% of that of the Al BM. In addition, all tensile specimens were fractured at the aluminum SZ.

The SZ hardness of the Al side increased from 0.578 to 0.63 GPa with an increase in the rotation speed from 300 to 500 RPM, due to the SZ grain refinement and the increase in SS fragments in the Al nugget. On the other hand, the SZ hardness of the SS side decreased from 5.038 to 3.6 GPa with an increase in the RPM.

## Figures and Tables

**Table 1:** Chemical composition (%) of the base metals.

Steel	C	Si	Mn	P	S	Ni	Cr	Mo	Fe	Al	Mg	Cu	Zn	Ti
AISI 316SS	0.04	0.89	1.8	0.03	0.02	12	17	2.7	Bal.	-	-	-	-	-
AL 6061-T6	-	0.61	-	-	-	-	0.3		0.17	98.2	0.58	0.28	0.01	0.012

**Table 2:** Mechanical properties of base materials.

Material	YS (MPa)	UTS (MPa)	A50 (%)
AISI 316	225	650	40
AL 6061-T6	250	290	14

**Table 3:** Friction stir welding parameters.

Sample No.	RPM	Travel Speed cm/min	Tilt Angle	AS	RS	Tool Offset
1	300	30	3°	AISI 316	AL6061	2 mm to Al side
2	400	20	3°	AISI 316	AL6061	2 mm to Al side
3	500	20	3°	AISI 316	AL6061	2 mm to Al side
4	600	20	3°	AISI 316	AL6061	2 mm to Al side

## Data Availability

Not applicable.
